# Effect of Supplemental Trace Mineral Source on Haircoat and Activity Levels in Senior Dogs

**DOI:** 10.3390/ani15050686

**Published:** 2025-02-26

**Authors:** Laura A. Amundson, Allison A. Millican, Erik Swensson, Mike L. McGilliard, Dana Tomlinson

**Affiliations:** 1 Zinpro Corporation, 10400 Viking Dr., Ste. 240, Eden Prairie, MN 55344, USA; amillican@zinpro.com (A.A.M.); eswensson@zinpro.com (E.S.); dtomlinson@zinpro.com (D.T.); 2 Department of Animal Sciences, Virginia Tech, Blacksburg, VA 24061, USA; mike.mcgilliard@gmail.com

**Keywords:** senior dogs, trace minerals, hair shedding, hair length, hair growth rate, hair quality, activity

## Abstract

Trace minerals (TMs) are pivotal for animal wellbeing and appearance. This study evaluated the effects of TM (Zn, Mn, Cu, and Fe) sources on senior dogs’ haircoat and activity. Dogs (mean age 9.16 yr) were fed a control diet (inorganic TM) for 14 d before being assigned to one of three treatments for 3 mo. Treatments included inorganic (Control; *n* = 10), amino acid-complexed (TMC; *n* = 17; ZINPRO^®^ ZnMet + Zinpro^®^ Availa^®^ Mins) or lysine and glutamic acid-complexed (TMC-LG; *n* = 16; Zinpro^®^ ProPath^®^) supplemental TM. Treatments were formulated to provide standard (Control—100 ppm Zn, 5 ppm Mn, 12 ppm Cu, 45 ppm Fe) or product-recommended (TMC and TMC-LG—100 ppm Zn, 25 ppm Mn, 7 ppm Cu, 45 ppm Fe) TM levels (DM basis). Hair length, regrowth, and shedding were measured along with activity. Dogs fed TMC (26.43 mm; 11.64 mm/mo) and TMC-LG (27.87 mm; 12.6 mm/mo) had significantly (*p* ≤ 0.05) longer hair that grew faster compared to the Control (18.93 mm; 9.14 mm/mo). At 3 mo TMC-LG-fed dogs had 0.38 g less shed hair, and TMC-fed dogs had 0.87 h more active time/d compared to the Control (*p* ≤ 0.05). Dogs fed TMC and TMC-LG had superior haircoat characteristics and activity compared to the Control.

## Simple Summary

Trace minerals (TMs) are essential for all living animals and vary in their bioavailability and effectiveness based on source. A randomized, controlled 90-day feeding study was conducted to compare the effect of inorganic vs. amino acid-complexed (organic) sources of Zn, Mn, Cu, and Fe on haircoat and activity levels in 43 healthy, senior dogs (Rottweilers, Golden Retrievers, and Labrador Retrievers). Dogs were fed diets supplemented with inorganic (Control), amino acid-complexed (TMC), or lysine and glutamic acid-complexed (TMC-LG) trace mineral sources. The results of the study showed that dogs fed TMC or TMC-LG had increased hair growth, superior hair quality, decreased hair shedding, and more active hours per day compared to those fed Control (inorganic) sources of trace minerals. Senior dogs are often afflicted with age-associated hair loss and decreased activity time. These results suggest that trace mineral nutrition from amino-acid complexed sources provides a management strategy to improve the appearance and vitality of senior dogs.

## 1. Introduction

Trace minerals are essential metal element nutrients needed in micro or trace amounts for all living animals. Trace minerals are essential for structural, physiological, and biochemical processes in animals, thereby making their inclusion in all animal diets necessary [1,2,3,4,5]. Several studies suggest the important role of trace minerals in the pathogenesis of disease in dogs [6,7,8]. Trace minerals are essential for healthy skin and coat across animal species. Through their roles in cellular integrity (Cu, Zn), structural support (Cu, Mn), and enzymatic processes (Zn, Mn, Cu, Fe), trace minerals are indispensable for epithelial tissue development and homeostasis; thus, directly influencing multiple physiological processes, including skin and coat health and overall health and wellbeing [1,2,3,4,5].

Canine health and longevity is of interest across all life stages but especially in senior dog populations. According to recent market research, the share of households in the United States with senior dogs was 53.5% in 2022, up from 41.6% in 2012 [9]. Advances in veterinary care and increasing owner attention to pets’ nutrition and overall wellbeing have increased life expectancy and improved quality of life of companion animals. As dogs age, there is an increased incidence of health problems such as diabetes, cardiac issues, and arthritis. In addition to medical problems, older dogs undergo physical changes in appearance where their skin becomes drier and flakier and their coats become rougher [10]. Senior dogs shed more hair, and their hair growth slows down. Other geriatric changes include a decline in activity levels [10]. Therefore, efforts to optimize senior dog nutrition are highly relevant.

Supplemental trace minerals are often added to companion animal diets [11] to balance inadequate ingredient trace mineral profiles. Still, many reports have shown that commercial pet foods have inadequate or excessive amounts of trace minerals [12,13,14,15]. Recent trends in companion animal nutrition, such as feeding raw food diets [16,17], have exacerbated some of these nutrient inadequacies. For example, raw food diets have been found to be deficient in some trace minerals, such as Zn and Cu [18]. Trace mineral bioavailability of natural ingredients is largely unknown. Therefore, even diets that may appear to meet nutrient requirements based on ingredient trace mineral concentrations are often supplemented with additional inorganic or organic minerals [2].

Trace mineral absorption depends on the elemental form and source of the mineral, both of which affect its bioavailability and utilization. Organic trace minerals are defined as trace metals that are chelated to carbon-containing molecules, such as amino acids, hydrolyzed proteins, or other carbon molecules. The Association of American Feed Control Officials (AAFCO) classifies organic trace minerals based on the chelating agents bound to the trace metal (metal–amino acid complexes, amino acid chelates, metal proteinates, and polysaccharide metal complexes) [19]. Organic trace mineral properties such as pH, stability, and bioavailability depend on the chelating agent as well as the production methods [20].

It is well known that inorganic and organic trace minerals are absorbed and utilized differently by the body [21]. Commercial dog foods are often supplemented with inorganic trace minerals. While this is an inexpensive way of incorporating trace minerals in complete diets, inorganic sources are often less bioavailable and less efficiently utilized compared to trace minerals from organic sources [21,22,23,24,25]; therefore, higher inclusion levels are often utilized to meet daily intake requirements, which could result in an imbalance of nutrients and increased toxicity risk [15,26,27].

In the last decade, there has been considerable interest in organic trace minerals in animal nutrition with several organic trace minerals commercially available [28]. Although organic trace minerals are generally considered more bioavailable, differences in chelating agents and production parameters offer sources of differentiation among the organic trace mineral classes [20]. Therefore, it is of interest to understand the efficacy of specific organic trace minerals to meet nutritional requirements for the health and longevity of dogs.

Trace mineral efficacy can be evaluated by relative bioavailability and phenotypic responses to dietary inclusion. In weanling puppies, zinc methionine was approximately three times more bioavailable compared to zinc oxide, thus indicating that it is a more readily available source of zinc to support the physiological needs of the puppies [29]. In a randomized, controlled, double-blind study conducted at two companion animal dermatology clinics, dogs with pruritic atopic dermatosis that were supplemented with zinc methionine plus biotin and essential fatty acids had reduced duration of atopy medicinal intervention compared to dogs supplemented with biotin and essential fatty acids alone [30]. Given the efficacy observed in these trials, we hypothesized that amino acid-complexed trace minerals could positively influence senior dogs.

The objective of the current study was to compare the effects of a diet formulated with industry standard sources (inorganic) and inclusion levels (Control) with diets formulated with two commercially available amino acid-complexed organic trace mineral sources (TMC and TMC-LG) on haircoat characteristics and overall wellbeing as measured by daily activity in senior dogs.

## 2. Materials and Methods

### 2.1. Study Design

The study was a randomized complete block design with dog as the experimental unit. The experimental timeline consisted of a 14-day acclimation period followed by a 90-day study period. The study was conducted at a USDA-inspected facility from June to October 2020. All dogs entering the study were fed the Control diet during the acclimation period to allow for diet adaptation and to avoid any physiological disruption from food changes. Additionally, this minimized residual effects of variable intake of trace minerals in the study population. Dogs were blocked by breed, sex, and age and randomly assigned to 1 of 3 dietary treatments, differing only in trace mineral source (Table 1 and Table 2). Dietary treatments consisted of 3 different supplemental (DM basis) trace mineral sources of Zn, Mn, Cu, and Fe: inorganic trace minerals (Control; 100 ppm Zn, 5 ppm Mn, 12 ppm Cu, 45 ppm Fe), a blend of amino acid-complexed trace minerals (TMC; Zinpro Corp. Eden Prairie, MN, USA; 100 ppm Zn, 25 ppm Mn, 7 ppm Cu, 45 ppm Fe), or lysine and glutamic acid-complexed trace minerals (TMC-LG; Zinpro Corp. Eden Prairie, MN; 100 ppm Zn, 25 ppm Mn, 7 ppm Cu, 45 ppm Fe). All dogs were fed their respective diets daily as the sole source of nutrition for the duration of the study. Animal technicians caring for the study animals were blinded to the dietary treatment throughout the study. Evaluations included general health observations; assessment of skin, hair, and coat; exercise/socialization reports; and activity monitoring using an accelerometer.

### 2.2. Experimental Animals

A total of 46 dogs (maximum available that met study criterion) were enrolled in the study at a USDA-certified research kennel. Enrolled dogs were chosen from the kennel’s available animals based on age (target population: senior dogs > 5 years of age) with breed and sex as further criteria. Two male Rottweilers, one in the Control group and one in the TMC-LG group, died during the study and were removed from the analysis. Neither death was related to dietary treatment; one was due to gastric dilation-volvulus, and the other was due to a cancerous tumor. Additionally, there was one dog in the TMC group that received additional zinc supplementation (20 ppm TMC-LG Zn) to nutritionally support absolution of a severe dermatologic flare coinciding with the study’s initiation and was therefore removed from analysis. The dog numbers in Table 3 reflect these removals. All other dogs were deemed healthy by the research kennel’s attending veterinarian.

Dogs enrolled in the study had an average age of 9.16 ± 0.28 years at the time of enrollment. There were 4 Rottweilers, 9 Golden Retrievers, and 30 Labrador Retrievers (mix of yellow and chocolate). Dogs were housed in pairs [2] to allow for normal socialization, unless prohibited due to dog temperament. Kennels (2.23 m^2^) were made of chain link fence with epoxy covered concrete floors and outside run access (7.43 m^2^). Dogs were offered assigned dietary treatments once a day (about 650 g/day) to maintain body weight throughout the study, and water was allowed ad libitum. Dogs were allowed to socialize and were exercised twice daily. Each dog had a FitBark^tm^ accelerometer attached to their collar to monitor daily activity. Dogs were fitted with FitBark^tm^ monitors one month prior to experimental diet implementation.

### 2.3. Study Diets

Treatment diets were commercially formulated to meet or exceed AAFCO Dog Food Nutrient Profiles for Adult Maintenance [19]. The only variable among treatment diets was the trace mineral premix, with respect to Zn, Mn, Cu, and Fe source. Diets were mixed and extruded according to industry standard manufacturing practices. Extrusion of all diets was completed at a single location on the same day, utilizing the same processing specification. Treatment diet composition and extruded diet nutrient analysis are shown in Table 1 and Table 2.

### 2.4. Evaluation of Hair Parameters

Hair parameters were evaluated at an area lateral to the spine, directly behind the shoulder. A 7.6 cm × 15.2 cm area was shaved with a 1.5 mm #10 blade and used to monitor hair length and hair growth rate from the base of the hair shaft at 3 different regions within the shaved area: front 1/3, mid-point, and back 1/3. Treatment means represent an average of the 3 regions. Hair samples were collected and placed in small sample bags with individual dog ID and date collected for observation under scanning electron microscope by TRI Princeton laboratory (Princeton, NJ, USA).

For evaluation of hair shedding, the dogs were brushed with a grooming brush (ten strokes) at a designated area at the top of the back/lumbar region guided by a template (~30 cm long). Hair was collected off the brush, weighed, photographed, and put in a sample bag with dog ID and date collected. The weekly kennel grooming protocol (bathing, grooming, ear cleaning) was discontinued and instead conducted monthly after experimental collections.

### 2.5. Evaluation of Activity Levels

Each dog had a FitBark^tm^ accelerometer attached to their collar, which monitored daily physical activity. Dogs were fitted with FitBark^tm^ monitors one month prior to experimental diet implementation. FitBark^tm^ monitors are commercially available and were used according to manufacturer’s instructions provided in the owner’s manual. Dog activity time was provided in the FitBark^tm^ output via proprietary calculations.

### 2.6. Statistical Analysis

Data were analyzed using a PROC GLIMMIX mixed model (SAS Inst., Int., Cary, NC, USA) with dog as the experimental unit; diet, breed, sex, and month as fixed effects; as well as the interactions for the animal characteristics, haircoat parameters, and activity. The July timepoint (start date) was used as a covariate for hair length, hair growth rate, shed hair, and active hours. Outliers were identified as observations beyond ±3 standard deviations of the mean. Statistical significance was determined at *p* ≤ 0.05 and trends considered when 0.05 ≤ *p* ≤ 0.10. Means are reported as LS means ± SEM (least square mean ± standard error of the mean). If a significant diet-by-month interaction was detected, a Tukey’s test was run to compare the dietary treatments within month.

## 3. Results

The study animal signalments are summarized in Table 4, respectively. Overall, there were no significant differences between treatment groups with respect to age, weight, and body condition score.

### 3.1. Haircoat Parameters

Given the different hair characteristics among breeds, it was not surprising there was an overall breed effect on hair length (*p* ≤ 0.05) with Golden Retrievers having the longest (29.02 ± 2.12 mm), followed by Rottweilers (22.90 ± 2.46 mm) and Labrador Retrievers (21.30 ± 1.02 mm). However, there was no treatment-by-breed interaction detected (*p =* 0.24); therefore, only treatment effects are reported in Table 4.

Trace mineral source significantly affected hair length (*p* ≤ 0.05) and hair growth rate (*p* ≤ 0.05; Table 4). Averaged across breeds and months, dogs in the TMC or TMC-LG groups had significantly longer hair than the Control, 7.50 mm (*p* ≤ 0.05) and 8.94 mm (*p* ≤ 0.05), respectively. Additionally, dogs in the TMC-LG group grew hair at a significantly faster rate (3.46 mm/month faster) than the dogs fed Control diets (*p* ≤ 0.05). One dog (Labrador Retriever) was removed (>3 SD of the mean) as a statistical outlier from the TMC group for hair length and hair growth rate analyses.

When averaged across breeds and months, there was no statistical difference in shed hair amount among the three groups (Table 4). Averaged across months, there was a treatment-by-breed trend detected (*p* ≤ 0.10). However, this response was driven by Rottweilers only. Among the treatment-by-breed comparisons, the only statistically significant difference was in Rottweilers fed Control compared to TMC-LG (*p* ≤ 0.05). Those fed TMC-LG had about 0.63 g less shed hair than those fed Control diets. It is important to note that there are only 4 Rottweilers included in this analysis, and therefore, caution should be taken when interpreting breed effects and interactions.

Average shed hair across breeds revealed a treatment-by-month trend (*p* ≤ 0.10). In month 3, dogs in the TMC-LG group had 0.38 g less shed hair compared to dogs fed the Control diets (*p* ≤ 0.05).

### 3.2. Activity Levels

Averaged across breeds and months, a treatment trend was detected in active hours (*p* ≤ 0.10) as measured by the FitBark^tm^ accelerometer attached to dogs’ collars (Table 5). Dogs in the TMC and TMC-LG group were active an average of 0.47 h/day more compared to Control-fed dogs. When averaged across breeds, a treatment-by-month trend was detected in active hours (*p* ≤ 0.10). In month 3, dogs in the TMC group were active 0.87 h/day more compared to Control-fed dogs (*p* ≤ 0.05).

### 3.3. Hair Quality

**Figure 1:**
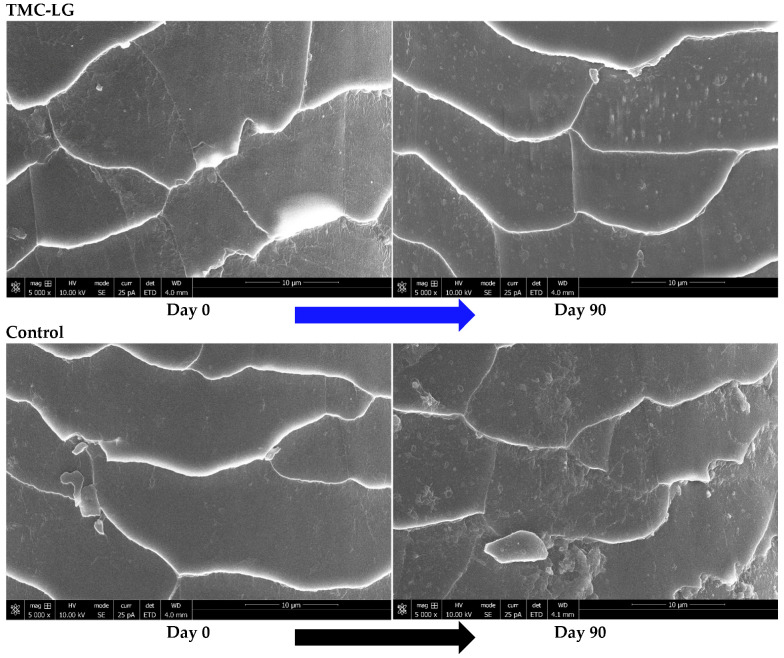
Impact of TMC-LG versus Control on hair shaft quality in dogs.

A representative hair shaft sample from the Control and TMC-LG groups was observed under a scanning electron microscope at day 0 and day 90 of the study. At day 0, hair from both groups appeared damaged with the edges of the hair cuticle raised (edges appear diffused and thicker). At day 90, however, the hair from the TMC-LG group had smooth edges (thinner, smoother, and less diffused), which did not appear raised in contrast to the Control group hair at day 90 (Figure 1).

## 4. Discussion

This study was designed to compare the effects of a diet formulated with industry standard sources (inorganic) and inclusion levels (Control) with diets formulated with recommended levels of two commercially available amino acid-complexed organic trace mineral sources (TMC and TMC-LG; Zinpro Corp. Eden Prairie, MN, USA) on haircoat characteristics and overall wellbeing as measured by daily activity in senior dogs. Results reported herein supported our hypothesis that the TMC and TMC-LG sources as part of a complete and balanced diet improved hair growth and quality, decreased hair shedding, and increased activity levels in senior dogs. To the best of the authors’ knowledge, this is the first study to evaluate the effect of organic trace minerals on haircoat characteristics and activity levels in senior dogs. The daily consumption of diets containing the three different sources of trace minerals for the entire duration of the study had no adverse effects on the physical appearance, food intake, body weight, body condition score, or general health of the animals.

Organic trace minerals are reported to be more bioavailable than inorganic forms, thereby allowing more minerals to be absorbed and effectively utilized by the animal [22,23,24,25], which should be evident in phenotypic traits. The enhanced mineral availability could be due to the protective effect of the ligand, thereby reducing interactions with other chelating agents while in the gut of the animal. This could prevent the formation of insoluble complexes such as phosphates and oxalates from rendering the mineral unavailable [31,32]. Chelated trace minerals generally have increased water and lipid solubility, thereby allowing absorption over a wide pH range, enhancing the minerals’ bioavailability [33]. However, caution should be heeded when grouping all organic trace mineral sources into one category due to the variations different chelating ligands produce [20], which could cause differences in bioavailability and bio-efficacy between organic trace mineral sources.

The amino acid-complexed trace mineral sources in the diets of the TMC (ZINPRO^®^ ZnMet + Zinpro^®^ Availa^®^ Mins; Zinpro Corp., Eden Prairie, MN, USA) and TMC-LG (Zinpro^®^ ProPath^®^; Zinpro Corp., Eden Prairie, MN, USA) groups are formulated to provide a 1:1 ratio of mineral to amino acids and are proven to utilize the amino acid transport system for absorption and metabolism [34]. This route of absorption prevents or reduces competition with other minerals for absorption by the body and protects from dietary antagonists, such as calcium, phytic acid, and folic acid. The uptake of these minerals allows them to remain in circulation longer for a more complete systemic delivery [35]. ZINPRO^®^ ZnMet and Zinpro^®^ Availa^®^ Mins (TMC) are complexed with methionine or a blend of several amino acids, whereas Zinpro^®^ ProPath^®^ (TMC-LG) minerals are complexed with lysine and glutamic acid only.

Dogs fed either TMC or TMC-LG had longer hair that grew faster compared to dogs fed Control diets. Although no statistically significant differences were detected between dogs fed TMC-LG and TMC, the TMC-LG group was numerically superior for hair length (1.44 mm longer) and hair growth rate (0.96 mm/mo faster) compared to TMC. In month 3, dogs fed TMC-LG had about 56% less shed hair compared to dogs fed Control diets and about a 35% reduction compared to dogs fed TMC. The effect of trace minerals on the body is not immediate [8] and may explain why it took a couple of months to observe a statistically significant reduction in shedding among treatment groups. Additionally, fall and spring are peak shedding seasons for dogs [36], and the observed reduction in shedding in October (Month 3) suggests that TMC-LG could be useful in managing seasonal shedding in dogs.

The TMC-LG source leverages two distinct amino acid transporters, the cationic (lysine) and anionic (glutamic acid). While glutamic acid is a non-essential amino acid for dogs, lysine is essential and must be provided in the diet [37]. This could explain the numerical improvement observed in the TMC-LG group compared to TMC.

Trace minerals play an important role in mammalian hair health [38,39,40,41]. Hair follicular cells have a high metabolism and rapid turnover, thereby requiring a constant supply of nutrients [42], particularly trace minerals that are part of cellular enzymes and transcriptions factors regulating immunity and antioxidant status [43]. Hydrogen peroxidase is crucial in catalyzing the breakdown of hydrogen peroxide to water and oxygen to defend against oxidative damage and is structurally dependent on iron [44]. Additional oxidative stress protection is provided by the cytosolic copper- and zinc-dependent and mitochondrial manganese-dependent antioxidants, superoxide dismutase 1 and 2, respectively [45]. Specific to companion animal skin and coat health, the primary protein in hair, nails, and the outermost layer of the epidermis is keratin. Zinc is essential for the catalytic, structural, and regulatory functions involved in tissue keratinization [46].

Our knowledge concerning the role of nutrition in hair follicle physiology is based on studies in humans, sheep, goats, and mice [38,39,40,41]. Whether this directly applies to dogs is not known. Canine hair anatomy differs from that of humans, goats, sheep, and mice [47]. In dogs, the telogen phase of the hair follicular cycle is also much longer than in humans, sheep, and mice, which have a longer anagen phase [48]. Despite these differences, the structure, composition, and high turnover rate of follicles remain the same across species [47,49] and therefore are likely affected by dietary nutrients in a similar manner.

As dogs age, they undergo changes in physical appearance, such as gray, dry, and dull coat; dry and flaky skin; loss of muscle mass; changes in body weight and condition; decreases in sensory health; in addition to acquiring systemic medical problems such as diabetes and cardiac or kidney disease [50]. Providing the best care to an aging pet that is healthy or with an age-related condition involves combining nutritional strategies, physical activity, and medical intervention, when needed. Regarding nutrition, there are no specific nutritional guidelines by AAFCO or FEDAIF as there is in the case of other life stages [51]. This is because the nutritional requirements could vary widely depending on whether the senior pet is generally healthy, has a single medical condition, or has several conflicting comorbidities. Regardless of the health condition of the senior pet, decreased hair growth or increased hair loss is often a problem either as part of the normal aging process or secondary to an age-associated condition. The results of the current study provide evidence to support micronutrient supplementation in senior dog diets with amino acid-complexed trace minerals could be a valuable approach in the nutritional management of a healthy haircoat.

Dogs fed TMC tended to be more active (~30 min/day), and dogs fed TMC-LG were numerically more active (~26 min/day) than those fed Control (inorganic) trace mineral sources in month 3 of the study. As observed with hair shedding, it took at least one month after the start of treatment to see an improvement in activity levels in dogs.

Several studies show the effectiveness of trace minerals on activity, energy levels, and athletic performance in humans [52,53], while studies in the veterinary field are limited. A couple of reports show the importance of organic trace minerals in exercised horses [54,55], the positive effects of which were attributed to the improved antioxidant status in these animals, thereby reducing oxidative stress and maintaining muscle health [55]. Enhanced glutathione peroxidase and superoxide dismutase, markers of antioxidant status, in pigs and reduced plasma malondialdehyde, a marker of oxidative stress, in broiler chickens were observed upon treatment with organic trace minerals [56,57]. These studies, however, did not measure animal activity levels. It is well established that oxidative stress leading to mitochondrial dysfunction is associated with aging and age-related functional losses due to systemic oxidative damage to macromolecules at the cellular level [58]. Studies have shown the benefits of dietary micronutrients in the delay of the aging process [59]. The current study was performed in an aging canine population, and the improvement in activity levels in this population when treated with amino acid-complexed trace minerals could be due to the improvement in their antioxidant status given the essential role Zn, Mn, Cu, and Fe play in maintaining proper oxidation status [44,45]. Although the mechanisms by which activity was increased in the current study is not fully understood, the results support the use of these amino-acid complexed trace minerals as part of a nutritionally complete diet to improve activity levels in senior dogs.

The authors recognize the study reported herein had some limitations. This study was designed to compare recommended inclusion levels of the TMC and TMC-LG products with standard industry trace mineral sources and inclusions, which caused a different concentration of Mn between the treatments. However, given the pivotal roles of Zn, Cu, and Fe discussed, it is unlikely that the difference in Mn inclusion was the sole reason for the observed treatment differences. The methodologies used to evaluate the variables of interest in this study provide practical data directly applicable to the companion animal industry but have not been validated. Blood samples could not be collected from the dogs during this study. Hence, the effect of these different trace mineral sources on the hematological and biochemical parameters of the dogs could not be ascertained. A study in growing lambs has shown that feeding organic trace minerals did not affect blood parameters [60]. Additionally, the dogs in this study did not show any visible signs of toxicosis during the feeding period. The lack of blood samples also prohibited exploration of potential cellular mechanisms (i.e., oxidative stress) to explain the observed differences between treatments. Another limitation is the limited data collected on hair quality. The data reported are limited to the subjective analysis of the SEM image. Additional research to elucidate the effect of trace mineral source on hair cycle and skin is warranted. Wholistically, there are other factors that could affect the coat and skin of senior dogs (sebum production, quality of keratin, thickness of hair strands, microflora of the skin or gut) that were not evaluated in this study. Lastly, the differences in activity could have also been affected by changes in bone and cartilage physiology, given the role that Zn, Mn, and Cu play in structural integrity. However, evaluation of bone and cartilage was not possible in this study. Further investigation of biochemical changes and objective measurements of mobility is warranted.

## 5. Conclusions

The study compared the effectiveness of two commercially available amino acid-complexed with inorganic trace mineral sources on hair growth, hair shedding, hair quality, and activity levels in senior dogs. Dogs fed TCM and TCM-LG (Zinpro Corp., Eden Prairie, MN, USA) showed faster hair growth, increased hair length, decreased shedding, and improved activity levels. The data support the use of TCM and TCM-LG mineral supplements as part of a complete and balanced diet to improve and maintain the overall wellbeing of senior dogs.

## Figures and Tables

**Table 1:** Treatment diet composition.

	Ingredient Inclusion, %		
Ingredient	Control	TMC	TMC-LG
Chicken meal, reg ash 15%	31.40	31.40	31.40
Milo	22.55	22.55	22.55
Corn	22.54	22.54	22.54
Wheat	21.89	21.80	21.88
Salt	0.625	0.625	0.625
Beet pulp	0.63	0.625	0.625
Vitamin premix ^1^	0.200	0.200	0.200
Control TM premix ^2^	0.115		
TMC TM premix ^3^		0.200	
TMC-LG TM premix ^4^			0.125
Choline Chloride 60%	0.050	0.050	0.050
Oxy-gon powder	0.015	0.015	0.015

^1^ Vitamin A, 17,163,000 IU/kg; vitamin D_3_, 920,000 IU/kg; vitamin E, 79,887 IU/kg; vitamin B_12_, 22 mg/kg; riboflavin, 4719 mg/kg; pantothenic acid, 12,186 mg/kg; niacin, 64,736 mg/kg; folic acid, 720 mg/kg; pyridoxine, 5537 mg/kg; thiamine, 14,252 mg/kg; biotin, 70 mg/kg. ^2^ CaCo_3_, ZnSO_4_ (Zn, 8.8%), FeSo_4_ (Fe, 3.9%), CuSO_4_ (Cu, 1.1%), MnO (Mn, 5684 ppm), Ca (IO_3_)_2_ (I, 1584 ppm), NaSeO_3_ (Se, 310 ppm). ^3^ ZINPRO^®^ ZnMet (Zn, 5.27%), Zinpro^®^ Availa^®^ Fe (Fe, 2.24%), Zinpro^®^ Availa^®^ Mn (Mn, 1.42%), Zinpro^®^ Availa^®^ Cu (Cu, 0.36%), Ca(IO_3_)_2_ (I, 873 ppm), NaSeO_3_ (Se, 175 ppm). ^4^ Zinpro^®^ ProPath^®^ Zn (Zn, 8.44%), Zinpro^®^ ProPath^®^ Fe (Fe, 3.57%), Zinpro^®^ ProPath^®^ Mn (Mn, 2.13%), Zinpro^®^ ProPath^®^ Cu (Cu, 0.59%), Ca(IO_3_)_2_ (I, 905 ppm), NaSeO_3_ (Se, 318 ppm).

**Table 2:** Treatment diet trace mineral (DM basis) and proximate analysis (As-fed basis) ^1^.

Nutrient		Control	TMC	TMC-LG
DM Basis				
	Zinc (Zn), ppm	159	158	158
	Iron (Fe), ppm	220	200	186
	Manganese (Mn), ppm	32	50	45
	Copper (Cu), ppm	19	16	16
	Iodine (I), ppm ^2^	1.8	1.7	1.1
	Selenium (Se), ppm ^2^	0.4	0.4	0.4
As-fed Basis				
	Dry matter, %	90.36	90.84	89.50
	Moisture, %	9.64	9.16	10.50
	Crude protein, %	25.50	25.80	25.60
	Fat, %	13.60	13.40	13.80
	Crude fiber, %	1.48	1.13	1.38
	Ash, %	6.17	5.81	6.22
	ME, kcal/kg^2^	3575	3606	3557
	Calcium (Ca), %	1.66	1.43	1.54
	Phosphorus (P), %	1.14	1.02	1.07

^1^ Proximate analysis—Midwest Laboratories (Omaha, NE). ^2^ Calculated.

**Table 3:** Animal characteristics for each group. Data are represented as absolute counts or as mean ± standard error ^1^.

Animal Characteristics		Group		
		Control	TMC	TMC-LG
Number of animals		10	17	16
Breed				
	Rottweilers	1	2	1
	Golden Retrievers	3	3	3
	Labrador Retreivers	6	12	12
Sex				
	Male (intact, neutered)	3 (2, 1)	7 (4, 3)	5 (4, 1)
	Female (intact, spayed)	7 (7, 0)	10 (8, 2)	11 (8, 3)
Mean age—start (yr)		9.33 ± 0.75	8.96 ± 0.59	9.08 ± 0.76
Mean weight—start (kg)		36.59 ± 1.85	34.50 ± 1.46	34.53 ± 1.88
Mean weight—end (kg)		37.47 ± 1.80	34.92 ± 1.42	33.85 ± 1.82
Body condition score—start		3.61 ± 0.30	3.68 ± 0.23	3.69 ± 0.30
Body condition score—end		3.58 ± 0.30	3.62 ± 0.23	3.55 ± 0.30

^1^ None of the age, weight, or body condition score parameters were statistically different between treatments.

**Table 4:** Trace mineral source effect on haircoat parameters ^1,2^.

	Control	ZINPRO + TMC	TMC-LG
Hair Length ^3^, mm	18.93 ± 1.91 ^a^	26.43 ± 1.54 ^b^	27.87 ± 1.89 ^b^
Hair Growth Rate ^3^, mm/mo	9.14 ± 0.97 ^a^	11.64 ± 0.82 ^a,b^	12.60 ± 1.05 ^b^
Shed Hair ^3^, g	0.43 ± 0.07	0.45 ± 0.07	0.29 ± 0.07
Treatment by month ^4,^*			
Month 1	0.36 ± 0.10	0.49 ± 0.10	0.34 ± 0.09
Month 2	0.26 ± 0.10	0.42 ± 0.10	0.24 ± 0.09
Month 3	0.67 ± 0.10 ^a^	0.45 ± 0.10 ^a,b^	0.29 ± 0.09 ^b^

^1^ Within a row, values with different letter superscripts are statistically different (*p* ≤ 0.05). Letters followed by * indicate a statistical trend (*p* ≤ 0.10). If no letter superscripts are present, there was not a statistically significant overall treatment effect. ^2^ Values are LS means ± SEM. ^3^ Treatment effect averaged across breeds and months. ^4^ Treatment trend by month, averaged across breeds, *p* ≤ 0.10.

**Table 5:** Trace mineral source effect on FitBark^tm^ parameters ^1,2,3^.

Active Hours, Hours/Day	Control	TMC	TMC-LG
Treatment ^4,5^	9.25 ± 0.18	9.72 ± 0.14	9.71 ± 0.17
Treatment by month ^6^			
Month 1	9.53 ± 0.21	9.66 ± 0.17	9.71 ± 0.20
Month 2	9.05 ± 0.21	9.47 ± 0.17	9.67 ± 0.20
Month 3	9.16 ± 0.21 ^a^	10.03 ± 0.17 ^b^	9.77 ± 0.20 ^a,b^

^1^ Within a row, values with different letter superscripts are statistically different (*p* ≤ 0.05). If no letter superscripts are present, there was not a statistically significant overall treatment effect. ^2^ Values are LS means ± SEM. ^3^ Activity %, Sleep %, and Health % are not included in this table as these are proprietary calculations by FitBark^tm^ and therefore interpretation of the data is incomplete. ^4^ Treatment effect averaged across breeds and months. ^5^ Overall treatment trend, *p* ≤ 0.10. ^6^ Treatment-by-month interaction, *p* ≤ 0.10.

## Data Availability

Restrictions apply to the availability of these data. Data were obtained by the study sponsor and may be available at the discretion of the study sponsor.

## Abbreviations

The following abbreviations are used in this manuscript:

MDPI	Multidisciplinary Digital Publishing Institute
AAFCO	American Association of Feed Control Officials
FEDIAF	European Pet food Industry Federation
SEM	Standard Error of the Mean
AOAC	Association of Official Agricultural Chemists
LS	Least Square
